# (1′*S*,12′*R*,13′*S*,17′*S*)-15′,15′-Dimethyl-1,2-dihydro-11′,14′,16′,18′-tetra­oxa-7′-aza­spiro­[indole-3,8′-penta­cyclo­[10.6.0.0^2,9^.0^3,7^.0^13,17^]octa­deca­ne]-2,10′-dione

**DOI:** 10.1107/S1600536813005436

**Published:** 2013-03-02

**Authors:** V. Sabari, R. Ponnusamy, R. Prasanna, R. Raghunathan, S. Aravindhan

**Affiliations:** a Department of Physics, Presidency College, Chennai 600 005, India; b Department of Computer Science & Engineering, Madha Engineering College, Kundrathur, Chennai 600 069, India; c Department of Organic Chemistry, University of Madras, Chennai 600 025, India

## Abstract

In the title compound, C_22_H_24_N_2_O_6_, the indole ring has a twist conformation and the tetra­hydro-2*H*-pyran-2-one ring a half-chair conformation. One of the pyrrolidine rings adopts an envelope conformation on the N atom, while the other has a twist conformation; the ‘butterfly’ angle between their mean planes is 62.98 (11)°. The dioxolane ring adopts a twist conformation and the tetra­hydro­furan ring has an envelope conformation on the C atom in the fused tetra­hydro-2*H*-pyran-2-one ring adjacent to the O atom of the tetra­hydro­furan ring. The ‘butterfly’ angle between the mean planes of these two five-membered rings is 69.14 (10)°. In the crystal, mol­ecules are linked by N—H⋯O hydrogen bonds, forming chains along the *a* axis.

## Related literature

For the biological activity of indole derivatives, see: Stevenson *et al.* (2000 ▶); Rajeswaran *et al.* (1999 ▶); Amal Raj *et al.* (2003 ▶). For a related structure, see: Jagadeesan *et al.* (2012 ▶).
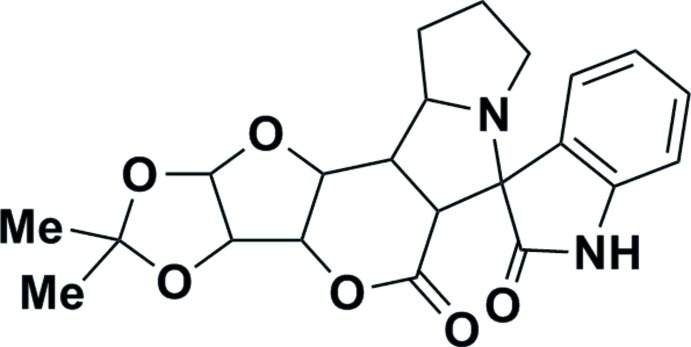


## Experimental

### 

#### Crystal data

C_22_H_24_N_2_O_6_
*M* *_r_* = 412.43
Orthorhombic, 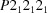

*a* = 9.2737 (5) Å
*b* = 11.6543 (8) Å
*c* = 18.8489 (14) Å
*V* = 2037.2 (2) Å^3^
*Z* = 4
Mo *K*α radiation
μ = 0.10 mm^−1^
*T* = 293 K
0.30 × 0.30 × 0.20 mm

#### Data collection

Bruker Kappa APEXII CCD diffractometer
Absorption correction: multi-scan (*SADABS*; Bruker 2008 ▶) *T*_min_ = 0.922, *T*_max_ = 0.947
21465 measured reflections
4717 independent reflections
3800 reflections with *I* > 2σ(*I*)
*R*_int_ = 0.036

#### Refinement

*R*[*F* ^2^ > 2σ(*F* ^2^)] = 0.037
*wR*(*F* ^2^) = 0.090
*S* = 1.04
4717 reflections
324 parameters
H atoms treated by a mixture of independent and constrained refinement
Δρ_max_ = 0.16 e Å^−3^
Δρ_min_ = −0.16 e Å^−3^

### 

Data collection: *APEX2* (Bruker, 2008 ▶); cell refinement: *SAINT* (Bruker, 2008 ▶); data reduction: *SAINT*; program(s) used to solve structure: *SHELXS97* (Sheldrick, 2008 ▶); program(s) used to refine structure: *SHELXL97* (Sheldrick, 2008 ▶); molecular graphics: *ORTEP-3 for Windows* (Farrugia, 2012 ▶); software used to prepare material for publication: *SHELXL97* and *PLATON* (Spek, 2009 ▶).

## Supplementary Material

Crystal structure: contains datablock(s) I, global. DOI: 10.1107/S1600536813005436/su2563sup1.cif

Structure factors: contains datablock(s) I. DOI: 10.1107/S1600536813005436/su2563Isup2.hkl

Supplementary material file. DOI: 10.1107/S1600536813005436/su2563Isup3.cml

Additional supplementary materials:  crystallographic information; 3D view; checkCIF report

## Figures and Tables

**Table 1:** Hydrogen-bond geometry (Å, °)

*D*—H⋯*A*	*D*—H	H⋯*A*	*D*⋯*A*	*D*—H⋯*A*
N1—H1⋯O2^i^	0.86	2.06	2.8849 (19)	161

Symmetry code: (i) 
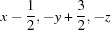
.

**Fig. 1.**
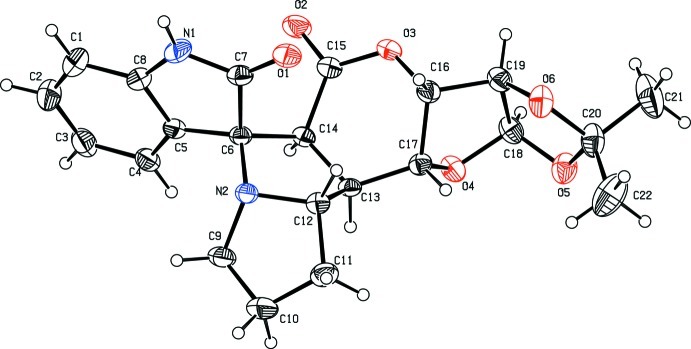
View of the molecular structure of the title molecule, showing the atom labelling. Displacement ellipsoids are drawn at the 30% probability level.

**Fig. 2.**
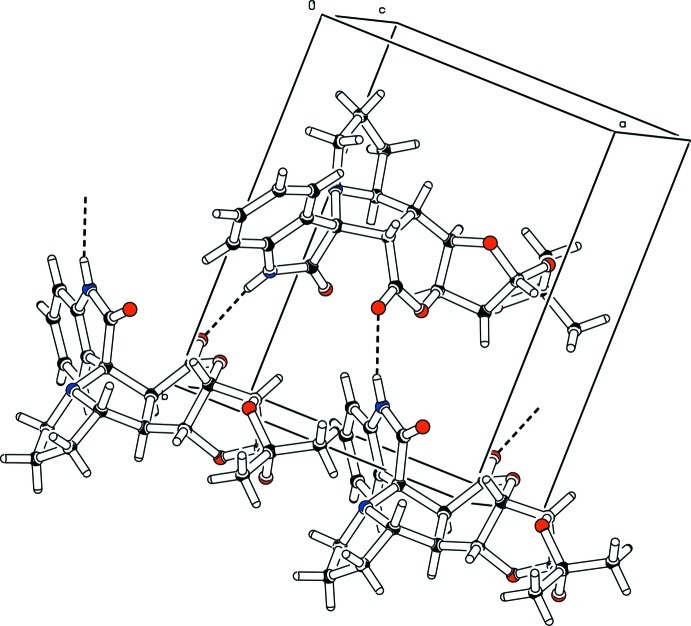
A view along the c axis of the crystal packing of the title compound, showing the N-H···O hydrogen bonds (dashed lines; see Table 1 for details).

## supplementary crystallographic information

### Comment

Indole compounds can be used as bioactive drugs (Stevenson *et al.*, 2000) and have also been proven to display high aldose reductase inhibitory activity (Rajeswaran *et al.*, 1999), and antimicrobial and antifungal activities (Amal Raj *et al.*, 2003).

The molecular structure of the title compound is shown in Fig. 1. The indole ring is essentially planar with the maximum deviation from planarity being 0.123 (2) Å for atom C7. Atom O1 deviates from the mean plane of the indole ring by 0.2095 (13) Å. The tetrahydro-2H-pyran-2-one ring (O3/C13-C17) has a half-chair conformation.

The five-membered pyrrolidine ring (N2/C9-C12) adopts an envelope conformation with atom N2 as the flap; it is 0.5267 (12) Å out of the mean plane formed by the other ring atoms. The other pyrrolidine ring (N2/C6/C12-C14) has a twist conformation on bond N2-C12; the "butter-fly" angle between their mean planes is 62.98 (11)°.

The dioxolane ring (O5/O6/C18-C20) adopts a twist conformation on bond O6-C20. The tetrahydrofuran ring (O4/C16-C19) adopts an envelope conformation with atom C17 deviating from the mean plane of the remaining ring atoms by 0.6286 (17) Å; the "butter-fly" angle between the ring mean planes is 69.14 (10) °.

In the crystal, molecules are linked via N-H···O hydrogen bonds forming chains propagating along the a axis direction (Table 1 and Fig. 2).

The title compound exhibits structural similarities with a related structure (Jagadeesan *et al.*, 2012).

### Experimental

A solution of 5,6-Dideoxy-l,2-*O*-isopropylidene-a-*D*-*xylo*-hept-5-enofuranurono-7,3-lactone (300 mg, 1.5 mmol), sarcosine (125 mg, 1.5 mmol) and isatin (210 mg, 1.5 mmol) were refluxed in dry toluene under a N_2_ atmosphere for 6–8 h at 383 K using a Dean-Stark apparatus. After the completion of the reaction as indicated by TLC, toluene was evaporated under reduced pressure. The crude product was washed with water and extracted with dichloromethane (4 × 20mL). The combined organic layers were dried (MgSO_4_) and filtered, concentrated in vacuum. The crude product was purified by column chromatography using hexane:EtOAc (7:3) mixture as eluent. On slow evaporation of the solvents colourless block-like crystals were obtained.

### Refinement

The methine and methylene H atoms were included in calculated positions freely refined. The remainder of the H atoms were included in calculated positions and allowed to ride on their parent atom: C—H = 0.93 - 0.96 Å, N-H = 0.86 Å, with *U*_iso_ = 1.5U_eq_(C-methyl), and = 1.2U_eq_(C,N) for other H atoms.

### Crystal data

**Table:**

C_22_H_24_N_2_O_6_	*F*(000) = 872
*M**_r_* = 412.43	*D*_x_ = 1.345 Mg m^−^^3^
Orthorhombic, *P*2_1_2_1_2_1_	Mo *K*α radiation, λ = 0.71073 Å
Hall symbol: P 2ac 2ab	Cell parameters from 8834 reflections
*a* = 9.2737 (5) Å	θ = 2.1–31.2°
*b* = 11.6543 (8) Å	µ = 0.10 mm^−^^1^
*c* = 18.8489 (14) Å	*T* = 293 K
*V* = 2037.2 (2) Å^3^	Block, colourless
*Z* = 4	0.30 × 0.30 × 0.20 mm

### Data collection

**Table:**

Bruker Kappa APEXII CCD diffractometer	4717 independent reflections
Radiation source: fine-focus sealed tube	3800 reflections with *I* > 2σ(*I*)
Graphite monochromator	*R*_int_ = 0.036
ω and φ scan	θ_max_ = 27.6°, θ_min_ = 2.1°
Absorption correction: multi-scan (*SADABS*; Bruker 2008)	*h* = −6→12
*T*_min_ = 0.922, *T*_max_ = 0.947	*k* = −15→14
21465 measured reflections	*l* = −24→24

### Refinement

**Table:**

Refinement on *F*^2^	Secondary atom site location: difference Fourier map
Least-squares matrix: full	Hydrogen site location: inferred from neighbouring sites
*R*[*F*^2^ > 2σ(*F*^2^)] = 0.037	H atoms treated by a mixture of independent and constrained refinement
*wR*(*F*^2^) = 0.090	*w* = 1/[σ^2^(*F*_o_^2^) + (0.0451*P*)^2^ + 0.1058*P*] where *P* = (*F*_o_^2^ + 2*F*_c_^2^)/3
*S* = 1.04	(Δ/σ)_max_ = 0.002
4717 reflections	Δρ_max_ = 0.16 e Å^−^^3^
324 parameters	Δρ_min_ = −0.16 e Å^−^^3^
0 restraints	Extinction correction: *SHELXL97* (Sheldrick, 2008), Fc^*^=kFc[1+0.001xFc^2^λ^3^/sin(2θ)]^-1/4^
Primary atom site location: structure-invariant direct methods	Extinction coefficient: 0.0043 (7)

### Special details

**Table:**

Geometry. All e.s.d.'s (except the e.s.d. in the dihedral angle between two l.s. planes) are estimated using the full covariance matrix. The cell e.s.d.'s are taken into account individually in the estimation of e.s.d.'s in distances, angles and torsion angles; correlations between e.s.d.'s in cell parameters are only used when they are defined by crystal symmetry. An approximate (isotropic) treatment of cell e.s.d.'s is used for estimating e.s.d.'s involving l.s. planes.
Refinement. Refinement of *F*^2^ against ALL reflections. The weighted *R*-factor *wR* and goodness of fit *S* are based on *F*^2^, conventional *R*-factors *R* are based on *F*, with *F* set to zero for negative *F*^2^. The threshold expression of *F*^2^ > σ(*F*^2^) is used only for calculating *R*-factors(gt) *etc*. and is not relevant to the choice of reflections for refinement. *R*-factors based on *F*^2^ are statistically about twice as large as those based on *F*, and *R*- factors based on ALL data will be even larger.

### Fractional atomic coordinates and isotropic or equivalent isotropic displacement parameters (Å^2^)

**Table:**

	*x*	*y*	*z*	*U*_iso_*/*U*_eq_	
H9A	0.090 (2)	0.2846 (19)	0.0396 (11)	0.064 (6)*	
H11A	0.384 (2)	0.2235 (18)	0.1817 (11)	0.059 (6)*	
H11B	0.251 (2)	0.278 (2)	0.2138 (12)	0.078 (7)*	
H10B	0.242 (3)	0.136 (3)	0.1028 (15)	0.109 (10)*	
H10A	0.115 (4)	0.178 (4)	0.1375 (19)	0.162 (15)*	
H12	0.3470 (19)	0.4460 (16)	0.1690 (10)	0.044 (5)*	
H9B	0.251 (2)	0.2576 (16)	0.0170 (10)	0.051 (5)*	
H17	0.6125 (17)	0.3803 (14)	0.1785 (9)	0.031 (4)*	
H16	0.5679 (19)	0.5796 (16)	0.1708 (10)	0.041 (5)*	
H14	0.4660 (17)	0.4184 (14)	−0.0175 (9)	0.034 (4)*	
H18	0.929 (2)	0.4543 (16)	0.0878 (10)	0.049 (5)*	
H13	0.4947 (16)	0.3027 (15)	0.0749 (8)	0.030 (4)*	
H19	0.8300 (18)	0.6200 (17)	0.1434 (9)	0.042 (5)*	
O4	0.74044 (11)	0.37954 (10)	0.09447 (6)	0.0428 (3)	
O1	0.30443 (14)	0.64064 (10)	0.11353 (7)	0.0494 (3)	
C13	0.48045 (16)	0.37792 (13)	0.08903 (9)	0.0321 (3)	
O2	0.51578 (14)	0.62812 (10)	−0.03068 (7)	0.0553 (4)	
N2	0.22433 (13)	0.39789 (11)	0.08173 (7)	0.0343 (3)	
O3	0.62164 (13)	0.59996 (10)	0.07089 (7)	0.0458 (3)	
O6	0.79932 (13)	0.52782 (12)	0.23306 (6)	0.0519 (3)	
O5	0.91693 (13)	0.38056 (12)	0.18080 (7)	0.0553 (4)	
N1	0.14101 (17)	0.64572 (13)	0.02193 (9)	0.0517 (4)	
H1	0.0942	0.7067	0.0334	0.062*	
C14	0.44834 (16)	0.45548 (13)	0.02581 (9)	0.0321 (3)	
C17	0.61096 (16)	0.41353 (14)	0.13092 (9)	0.0346 (3)	
C5	0.20045 (16)	0.48417 (13)	−0.03945 (9)	0.0364 (4)	
C12	0.33988 (16)	0.37913 (14)	0.13385 (9)	0.0353 (4)	
C7	0.24724 (18)	0.59935 (14)	0.06148 (9)	0.0392 (4)	
C11	0.3016 (2)	0.26639 (17)	0.17006 (12)	0.0498 (5)	
C16	0.63188 (18)	0.54190 (14)	0.13806 (9)	0.0382 (4)	
C8	0.11618 (19)	0.58297 (15)	−0.03980 (10)	0.0465 (4)	
C4	0.20154 (18)	0.41338 (15)	−0.09800 (9)	0.0435 (4)	
H4	0.2601	0.3486	−0.0991	0.052*	
C6	0.28163 (15)	0.47975 (12)	0.02961 (8)	0.0329 (3)	
C15	0.53006 (18)	0.56665 (14)	0.02019 (9)	0.0386 (4)	
C9	0.1869 (2)	0.28246 (15)	0.05469 (11)	0.0471 (4)	
C19	0.78866 (19)	0.54907 (17)	0.15952 (10)	0.0439 (4)	
C20	0.91327 (19)	0.4474 (2)	0.24389 (11)	0.0555 (5)	
C1	0.0264 (2)	0.6093 (2)	−0.09595 (12)	0.0644 (6)	
H1A	−0.0322	0.6740	−0.0951	0.077*	
C3	0.1137 (2)	0.44023 (19)	−0.15530 (10)	0.0558 (5)	
H3	0.1136	0.3933	−0.1952	0.067*	
C18	0.85583 (18)	0.44176 (16)	0.12504 (10)	0.0437 (4)	
C2	0.0269 (2)	0.5360 (2)	−0.15326 (12)	0.0688 (6)	
H2	−0.0330	0.5516	−0.1916	0.083*	
C10	0.2036 (3)	0.2056 (2)	0.11837 (16)	0.0706 (7)	
C21	1.0539 (2)	0.5104 (3)	0.25265 (17)	0.0961 (10)	
H21B	1.1302	0.4561	0.2602	0.144*	
H21C	1.0476	0.5610	0.2927	0.144*	
H21A	1.0736	0.5542	0.2106	0.144*	
C22	0.8736 (3)	0.3709 (3)	0.30471 (13)	0.0978 (10)	
H22A	0.9498	0.3167	0.3128	0.147*	
H22C	0.7862	0.3306	0.2937	0.147*	
H22B	0.8595	0.4165	0.3466	0.147*	

### Atomic displacement parameters (Å^2^)

**Table:**

	*U*^11^	*U*^22^	*U*^33^	*U*^12^	*U*^13^	*U*^23^
O4	0.0336 (5)	0.0432 (6)	0.0518 (7)	0.0007 (5)	0.0049 (5)	−0.0152 (6)
O1	0.0596 (7)	0.0338 (6)	0.0546 (8)	0.0031 (5)	−0.0002 (6)	−0.0115 (6)
C13	0.0352 (7)	0.0216 (8)	0.0394 (9)	0.0007 (6)	0.0028 (6)	−0.0012 (7)
O2	0.0696 (8)	0.0466 (7)	0.0496 (8)	−0.0215 (6)	−0.0061 (6)	0.0149 (6)
N2	0.0331 (6)	0.0280 (6)	0.0419 (7)	−0.0016 (5)	0.0016 (5)	0.0005 (6)
O3	0.0509 (7)	0.0329 (6)	0.0535 (7)	−0.0120 (6)	−0.0089 (6)	0.0030 (6)
O6	0.0454 (7)	0.0664 (9)	0.0441 (7)	0.0126 (6)	−0.0043 (5)	−0.0161 (6)
O5	0.0511 (7)	0.0602 (9)	0.0545 (8)	0.0143 (6)	−0.0071 (6)	−0.0121 (7)
N1	0.0562 (9)	0.0333 (8)	0.0657 (10)	0.0160 (7)	−0.0051 (8)	−0.0021 (8)
C14	0.0336 (7)	0.0280 (8)	0.0346 (8)	−0.0032 (6)	0.0036 (6)	−0.0044 (7)
C17	0.0335 (8)	0.0327 (8)	0.0375 (9)	0.0038 (6)	0.0040 (7)	−0.0002 (7)
C5	0.0356 (8)	0.0292 (8)	0.0444 (9)	−0.0045 (6)	−0.0007 (7)	0.0028 (7)
C12	0.0367 (8)	0.0330 (8)	0.0362 (9)	0.0007 (7)	0.0041 (6)	0.0002 (7)
C7	0.0423 (8)	0.0278 (8)	0.0474 (10)	0.0007 (7)	0.0057 (8)	−0.0012 (7)
C11	0.0486 (10)	0.0453 (11)	0.0554 (12)	−0.0017 (9)	0.0066 (9)	0.0142 (9)
C16	0.0405 (8)	0.0339 (9)	0.0402 (9)	−0.0007 (7)	−0.0011 (7)	−0.0050 (7)
C8	0.0477 (9)	0.0358 (9)	0.0559 (11)	0.0019 (8)	−0.0069 (9)	0.0064 (9)
C4	0.0430 (9)	0.0398 (9)	0.0478 (10)	−0.0094 (8)	−0.0006 (8)	−0.0002 (8)
C6	0.0336 (7)	0.0245 (7)	0.0404 (9)	−0.0003 (6)	0.0016 (6)	−0.0007 (7)
C15	0.0412 (8)	0.0328 (8)	0.0418 (9)	−0.0052 (7)	0.0030 (7)	0.0011 (8)
C9	0.0533 (11)	0.0297 (9)	0.0584 (12)	−0.0073 (8)	0.0010 (10)	−0.0008 (8)
C19	0.0434 (9)	0.0417 (10)	0.0466 (10)	−0.0042 (8)	−0.0017 (8)	−0.0079 (8)
C20	0.0406 (9)	0.0743 (14)	0.0518 (11)	0.0117 (9)	−0.0063 (8)	−0.0156 (11)
C1	0.0631 (12)	0.0561 (12)	0.0740 (15)	0.0123 (11)	−0.0187 (11)	0.0104 (11)
C3	0.0543 (11)	0.0626 (13)	0.0504 (11)	−0.0167 (10)	−0.0088 (9)	−0.0014 (10)
C18	0.0340 (8)	0.0526 (11)	0.0445 (10)	−0.0053 (7)	0.0061 (8)	−0.0099 (9)
C2	0.0627 (13)	0.0754 (15)	0.0684 (15)	−0.0042 (12)	−0.0246 (11)	0.0161 (13)
C10	0.0868 (17)	0.0419 (12)	0.0830 (17)	−0.0201 (12)	−0.0164 (14)	0.0179 (12)
C21	0.0457 (12)	0.119 (2)	0.123 (2)	0.0087 (14)	−0.0179 (13)	−0.057 (2)
C22	0.108 (2)	0.126 (3)	0.0594 (15)	0.040 (2)	−0.0009 (14)	0.0199 (17)

### Geometric parameters (Å, º)

**Table:**

O4—C18	1.415 (2)	C7—C6	1.551 (2)
O4—C17	1.4390 (18)	C11—C10	1.509 (3)
O1—C7	1.215 (2)	C11—H11A	0.94 (2)
C13—C17	1.503 (2)	C11—H11B	0.96 (2)
C13—C14	1.525 (2)	C16—C19	1.511 (2)
C13—C12	1.553 (2)	C16—H16	0.962 (19)
C13—H13	0.925 (17)	C8—C1	1.381 (3)
O2—C15	1.204 (2)	C4—C3	1.388 (3)
N2—C6	1.469 (2)	C4—H4	0.9300
N2—C12	1.470 (2)	C9—C10	1.506 (3)
N2—C9	1.480 (2)	C9—H9A	0.95 (2)
O3—C15	1.336 (2)	C9—H9B	0.97 (2)
O3—C16	1.439 (2)	C19—C18	1.541 (2)
O6—C19	1.411 (2)	C19—H19	0.961 (19)
O6—C20	1.427 (2)	C20—C22	1.498 (3)
O5—C18	1.391 (2)	C20—C21	1.505 (3)
O5—C20	1.422 (2)	C1—C2	1.377 (3)
N1—C7	1.348 (2)	C1—H1A	0.9300
N1—C8	1.393 (2)	C3—C2	1.376 (3)
N1—H1	0.8600	C3—H3	0.9300
C14—C15	1.505 (2)	C18—H18	0.985 (19)
C14—C6	1.573 (2)	C2—H2	0.9300
C14—H14	0.938 (17)	C10—H10B	0.93 (3)
C17—C16	1.515 (2)	C10—H10A	0.95 (4)
C17—H17	0.977 (17)	C21—H21B	0.9600
C5—C4	1.378 (2)	C21—H21C	0.9600
C5—C8	1.392 (2)	C21—H21A	0.9600
C5—C6	1.505 (2)	C22—H22A	0.9600
C12—C11	1.523 (2)	C22—H22C	0.9600
C12—H12	1.026 (19)	C22—H22B	0.9600
			
C18—O4—C17	107.19 (11)	N2—C6—C7	104.49 (12)
C17—C13—C14	113.82 (13)	C5—C6—C7	101.62 (13)
C17—C13—C12	112.80 (14)	N2—C6—C14	105.62 (12)
C14—C13—C12	104.82 (12)	C5—C6—C14	117.28 (13)
C17—C13—H13	107.3 (10)	C7—C6—C14	112.42 (12)
C14—C13—H13	111.4 (10)	O2—C15—O3	117.81 (14)
C12—C13—H13	106.6 (10)	O2—C15—C14	120.85 (15)
C6—N2—C12	106.25 (11)	O3—C15—C14	121.33 (15)
C6—N2—C9	116.41 (13)	N2—C9—C10	104.02 (17)
C12—N2—C9	105.42 (13)	N2—C9—H9A	107.6 (14)
C15—O3—C16	122.32 (12)	C10—C9—H9A	110.7 (13)
C19—O6—C20	107.92 (14)	N2—C9—H9B	112.2 (11)
C18—O5—C20	109.96 (15)	C10—C9—H9B	110.0 (11)
C7—N1—C8	111.83 (14)	H9A—C9—H9B	112.0 (17)
C7—N1—H1	124.1	O6—C19—C16	108.69 (15)
C8—N1—H1	124.1	O6—C19—C18	104.10 (15)
C15—C14—C13	117.84 (13)	C16—C19—C18	103.36 (14)
C15—C14—C6	110.08 (13)	O6—C19—H19	115.7 (10)
C13—C14—C6	105.24 (12)	C16—C19—H19	110.3 (10)
C15—C14—H14	104.3 (10)	C18—C19—H19	113.9 (10)
C13—C14—H14	111.9 (10)	O5—C20—O6	104.94 (14)
C6—C14—H14	107.1 (10)	O5—C20—C22	108.7 (2)
O4—C17—C13	110.19 (13)	O6—C20—C22	108.59 (17)
O4—C17—C16	101.97 (13)	O5—C20—C21	109.76 (17)
C13—C17—C16	114.99 (14)	O6—C20—C21	109.69 (19)
O4—C17—H17	108.4 (9)	C22—C20—C21	114.7 (2)
C13—C17—H17	112.6 (9)	C2—C1—C8	117.46 (19)
C16—C17—H17	108.0 (10)	C2—C1—H1A	121.3
C4—C5—C8	119.72 (15)	C8—C1—H1A	121.3
C4—C5—C6	131.97 (14)	C2—C3—C4	120.3 (2)
C8—C5—C6	108.26 (14)	C2—C3—H3	119.9
N2—C12—C11	104.95 (14)	C4—C3—H3	119.9
N2—C12—C13	104.46 (12)	O5—C18—O4	110.66 (15)
C11—C12—C13	115.57 (14)	O5—C18—C19	105.19 (14)
N2—C12—H12	111.5 (10)	O4—C18—C19	106.37 (13)
C11—C12—H12	112.4 (10)	O5—C18—H18	109.7 (11)
C13—C12—H12	107.7 (10)	O4—C18—H18	107.7 (11)
O1—C7—N1	127.35 (15)	C19—C18—H18	117.2 (11)
O1—C7—C6	125.38 (15)	C3—C2—C1	121.80 (19)
N1—C7—C6	107.24 (14)	C3—C2—H2	119.1
C10—C11—C12	104.84 (17)	C1—C2—H2	119.1
C10—C11—H11A	113.1 (13)	C9—C10—C11	107.29 (16)
C12—C11—H11A	111.9 (13)	C9—C10—H10B	107.9 (18)
C10—C11—H11B	108.9 (14)	C11—C10—H10B	112.7 (19)
C12—C11—H11B	112.5 (15)	C9—C10—H10A	114 (2)
H11A—C11—H11B	105.7 (18)	C11—C10—H10A	116 (2)
O3—C16—C19	105.85 (14)	H10B—C10—H10A	99 (3)
O3—C16—C17	112.20 (14)	C20—C21—H21B	109.5
C19—C16—C17	101.63 (14)	C20—C21—H21C	109.5
O3—C16—H16	108.0 (11)	H21B—C21—H21C	109.5
C19—C16—H16	113.4 (10)	C20—C21—H21A	109.5
C17—C16—H16	115.4 (11)	H21B—C21—H21A	109.5
C1—C8—C5	121.73 (18)	H21C—C21—H21A	109.5
C1—C8—N1	128.53 (18)	C20—C22—H22A	109.5
C5—C8—N1	109.73 (15)	C20—C22—H22C	109.5
C5—C4—C3	118.93 (17)	H22A—C22—H22C	109.5
C5—C4—H4	120.5	C20—C22—H22B	109.5
C3—C4—H4	120.5	H22A—C22—H22B	109.5
N2—C6—C5	114.83 (12)	H22C—C22—H22B	109.5

### Hydrogen-bond geometry (Å, º)

**Table:**

*D*—H···*A*	*D*—H	H···*A*	*D*···*A*	*D*—H···*A*
N1—H1···O2^i^	0.86	2.06	2.8849 (19)	161

Symmetry code: (i) *x*−1/2, −*y*+3/2, −*z*.
